# 

**Published:** 1858-03

**Authors:** 


### RECEIPTS ON SUBSCRIPTIONS,

Up to March 15th, 1858.

### ILLINOIS.

| | | | | |
|---|---|---|---|---|
| Dr. | T. D. Fitch, | vol. 6, | | $2 00 |
| " | H. Newell, | " 4, 5 & 6, | | 6 00 |
| " | Little & Lewis, | " 4, 5 & 6, | | 6 00 |
| " | James Kelly, | " 1, | 1858, | 2 00 |
| " | F. Ehrhardt, | " 6, | | 2 00 |
| " | L. Hale, | " 5 & 6, | | 5 00 |
| " | L. Clark, | " 1, | 1858, | 2 00 |
| " | W. W. Walker, | " 6, | | 2 00 |
| " | John Crim, | " 1, | 1858, | 2 00 |
| " | P. A. Allaire, | " 1, | " | 2 00 |
| " | J. W. Trabue, | " 1, | " | 2 00 |
| " | D. W. Young, | " 1, | " | 2 00 |
| " | J. G. Tilden, | " 6, | | 2 00 |
| " | J. P. H. Stevenson, | " 6, | | 2 00 |
| " | J. A. Roman, | " 1, | 1858, | 2 00 |
| " | W. R. Griswold, | " 1, | " | 2 00 |
| " | Wm. Law, | " 4, 5 & 6, | | 6 00 |
| " | David Prince, | " 6, | | 2 00 |
| " | P. A. Rosenberger, | " 6 & 1, | | 4 00 |

### WISCONSIN.

| | | | | |
|---|---|---|---|---|
| Dr. | G. W. Lee, | vol. 6, | | $2 00 |
| " | James Robie, | " 1, | 1858, | 2 00 |
| " | W. W. Lake, | " 5 & 6, | | 4 00 |
| " | S. A. Pease, | " 5, 6 & 1, | | 5 00 |
| " | Hays McKinlay, | " 6 & 1, | | 4 00 |
| " | H D. Adams, | " 6, | | 2 00 |
| " | B. O. Reynolds, | " 6, | | 2 00 |

### IOWA.

| | | | | |
|---|---|---|---|---|
| Dr. | J. H. Foster, | vol. 1, | 1858, | $2 00 |
| " | F. W. Coolidge, | " 5, 6 & 1, | | 6 00 |
| " | Ephraim Clifford, | " 6, | | 2 00 |
| " | A. A. & H. C. Rawson, | vol. 1, | 1858, | 2 00 |
| " | Macy & Mullinix, | " 1, | " | 2 00 |

### INDIANA.

| | | | | |
|---|---|---|---|---|
| Dr. | N. H. Canady, | vol. 1, | 1858, | $2 00 |
| " | S. F. Messner, | " 4, 5 & 6, | | 6 00 |
| " | J. Q. A. Banta, | " 6, | | 2 00 |
| " | L. T. Langdon, | " 6, | | 2 00 |
| " | J. Dancer, | " 1, | 1858, | 2 00 |
| " | S. F. McCarthy, | " 5 & 6, | | 4 00 |
| " | M. H. Bonnel, | " 1, | 1858, | 2 00 |
| " | R. Knappstadt, | " 1, | " | 2 00 |
| " | S. W. Purviance, | " 5 & 6, | | 4 00 |
| " | A. J. Minnick, | " 6, | | 2 00 |
| " | Long & Ellis, | " 1, | 1858, | 2 00 |

### MISSOURI.

| | | | | |
|---|---|---|---|---|
| Dr. | E. Jennison, | vol. 6, | | $2 00 |
| " | J. J. Fail, | " 1, | 1858, | 2 00 |
| " | A. J. Wade, | " 5, | | 2 00 |

### MICHIGAN.

| | | | | |
|---|---|---|---|---|
| Dr. | S. P. Root, | vols. 5 & 6, | | $4 00 |

### KANSAS TERRITORY.

| | | | | |
|---|---|---|---|---|
| Dr. | M. F. Holoday, | vol. 1, | 1858, | $2 00 |

